# Phosphorylated Nucleolin Interacts with Translationally Controlled Tumor Protein during Mitosis and with Oct4 during Interphase in ES Cells

**DOI:** 10.1371/journal.pone.0013678

**Published:** 2010-10-27

**Authors:** Helena Johansson, Frida Svensson, Rikard Runnberg, Tomas Simonsson, Stina Simonsson

**Affiliations:** Department of Medical Biochemistry and Cell Biology, Institute of Biomedicine, University of Gothenburg, Gothenburg, Sweden; Brigham and Women's Hospital, United States of America

## Abstract

**Background:**

Reprogramming of somatic cells for derivation of either embryonic stem (ES) cells, by somatic cell nuclear transfer (SCNT), or ES-like cells, by induced pluripotent stem (iPS) cell procedure, provides potential routes toward non-immunogenic cell replacement therapies. Nucleolar proteins serve as markers for activation of embryonic genes, whose expression is crucial for successful reprogramming. Although Nucleolin (Ncl) is one of the most abundant nucleolar proteins, its interaction partners in ES cells have remained unidentified.

**Methodology:**

Here we explored novel Ncl-interacting proteins using *in situ* proximity ligation assay (PLA), colocalization and immunoprecipitation (IP) in ES cells.

**Principal Findings:**

We found that phosphorylated Ncl (Ncl-P) interacted with translationally controlled tumor protein (Tpt1) in murine ES cells. The Ncl-P/Tpt1 complex peaked during mitosis and was reduced upon retinoic acid induced differentiation, signifying a role in cell proliferation. In addition, we showed that Ncl-P interacted with the transcription factor Oct4 during interphase in human as well as murine ES cells, indicating of a role in transcription. The Ncl-P/Oct4 complex peaked during early stages of spontaneous human ES cell differentiation and may thus be involved in the initial differentiation event(s) of mammalian development.

**Conclusions:**

Here we described two novel protein-protein interactions in ES cells, which give us further insight into the complex network of interacting proteins in pluripotent cells.

## Introduction

Nuclear reprogramming of somatic cells is a promising route in cell replacement therapy that can be used to replace or restore normal function of damaged cells. The ultimate approach is to reprogram the patient's own cell, which would avoid immunosuppression. The molecular mechanisms of nuclear reprogramming are still unsolved although recent reports have shown that reprogramming of human somatic cells can be achieved *in vitro* by retroviral expression of four transcription factors creating induced pluripotent stem (iPS) cells, which are comparable to ES cells [1], [2], [3], [4]. One factor has been proven essential for successful iPS cell creation i.e. Oct4, which is an established ES cell [5], [6] and potent nuclear reprogramming factor [7], [8], [9]. Successful reprogramming of somatic cells requires proper embryonic genome activation. In rhesus monkey, the major embryonic genome activation is thought to occur between the six- and eight-cell stages, which coincide with the timing of nucleogenesis [10]. The nucleolus may therefore serve as a marker for embryonic genome activation.

Nucleolin (Ncl, also denoted C23), one of the most abundant non-ribosomal proteins in the nucleoli, is a multifunctional protein [11], which belongs to a large family of RNA binding proteins and is a substrate to several kinases. Extensive phosphorylation occurs during interphase on serine residues by CK2 [12], while cdc2 phosphorylate threonine residues during mitosis [13], and these phosphorylation patterns regulate Ncl functions and localization during the cell cycle.

Ncl is functionally hyperactive in rapidly dividing cells compared to nondividing cells, and high levels of Ncl are found in tumors [14] and other rapidly dividing cells such as ES cells [15], [16], indicating several important functions during cell proliferation. A unique regulatory mechanism for Ncl expression has been revealed; Ncl has increased stability in proliferating cells by inhibiting its self-cleaving activity [17]. Down-regulation experiments using RNA-interference has proven that Ncl is essential for cell division, given that absence of Ncl results in prolonged cell cycle with misaligned chromosomes, defects in spindle organization [18], growth arrest and increased apoptosis [19]. Ncl has also been reported to have a cell cycle-controlled interaction with the prototypical tumor suppressor Retinoblastoma protein (Rb), only occurring during G1 when Rb is unphosphorylated and this interaction inhibits the DNA binding function of Ncl and determines its cellular localization [20]. ES cells possess a unique cell cycle quite different from committed cells [21], especially G1 is shortened and Rb is hyperphosphorylated during the whole cell cycle, indicating that Ncl should not be influenced or in a complex with Rb in ES cells.

Ncl is also described to interact with or be part of several transcription factor complexes, one of them being the B cell-specific transcription factor and switch region binding protein, LR1 [22]. Ncl has been shown to activate endogenous *Bcl-2* and CD34 gene expression in CD34 positive hematopoietic cells by a direct sequence-specific interaction with the CD34 promoter region and is thus involved in the maintenance of these progenitor/stem cells in hematopoiesis [23].

Even though Ncl is expressed at high levels in ES cells, not much is known about its specific role or physical interaction network in this cell type; therefore we explored Ncl in ES cells, starting with a search for new interaction partners. In current study, we show that Ncl interacts individually with translationally controlled tumor protein (Tpt1) and Oct4 in a cell cycle dependent manner, and both complexes require phosphorylation of Ncl.

## Results

### Identification of Ncl as a possible Tpt1 interaction partner in ES cells

**Figure 1:**
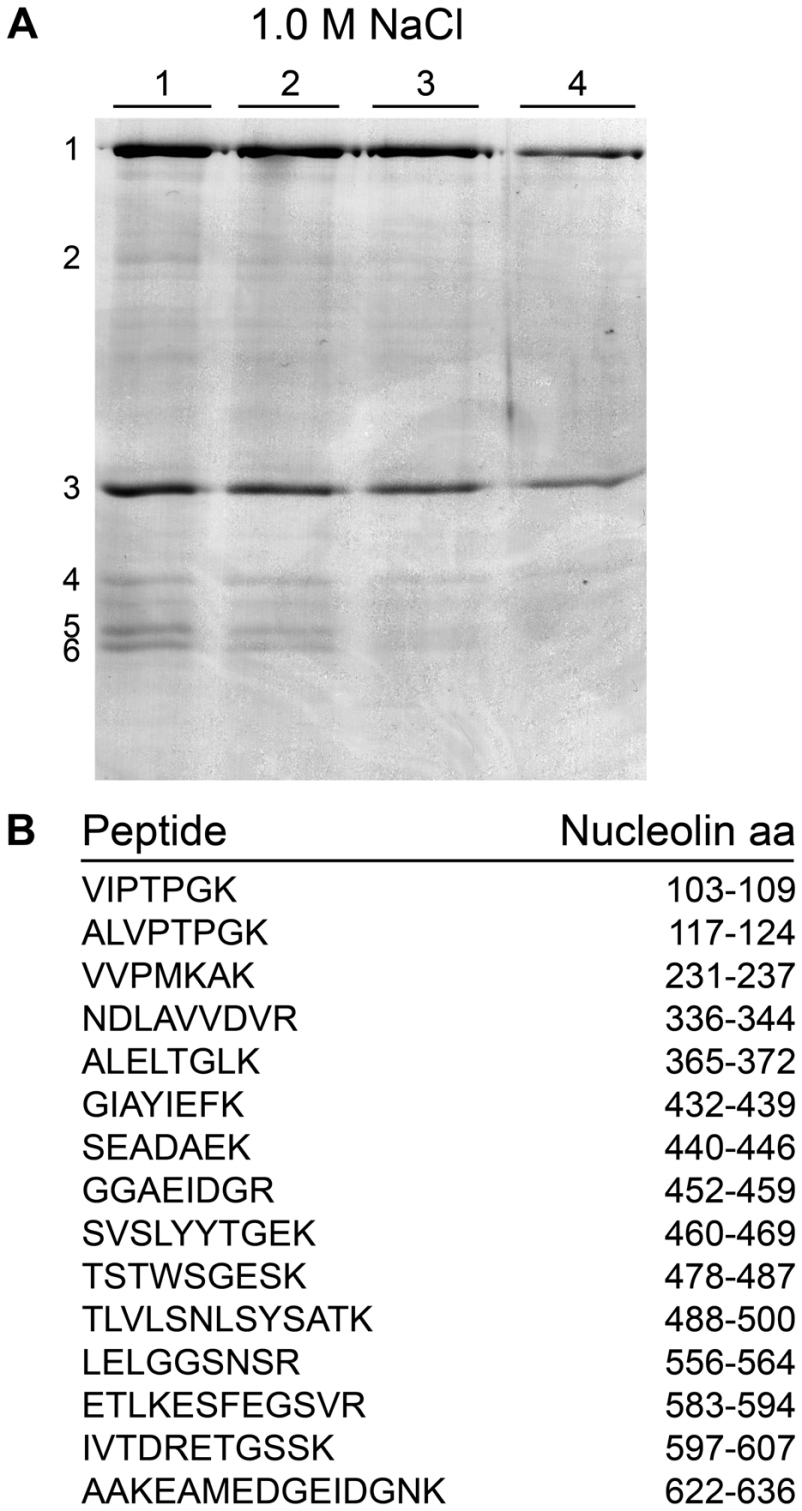
Identification of Ncl as a possible Tpt1 interaction partner. (A) Whole ES cell extract was incubated with sepharose-linked Tpt1 and bound proteins were eluted with increasing ionic strength. Lane 1–4 represents different fractions from the 1.0 M NaCl elute. Bands of interest (numbered 1–6, lane 1) were excised and identified by peptide sequencing, which gave the following results: **1**. Myosin heavy polypeptide 9; **2**. Nucleolin; **3**. Actin, cytoplasmic type 8, γ-actin, β-actin; **4**. Ribosomal protein L10, Tropomyosin; **5**. H1d, H1e, H1b, Ribosomal protein small subunit, Tropomyosin; and **6**. H1a, Tropomyosin, ribosomal protein L8. (B) Nano-LC FT-ICR mass spectrometry uniquely identified band number 2 as nucleolin. Sequences of 15 peptide fragments from the excised band are shown.

We have previously reported that translationally controlled tumor protein (Tpt1) physically interacts with nucleophosmin (Npm1) in murine ES cells in a cell cycle specific manner [24]. In the same screen, using recombinant Tpt1 covalently linked to cyanogen bromide activated sepharose beads, incubated with total ES cell extract, several proteins were eluted with increasing ionic strength. The eluted fractions at 1.0 M NaCl were separated by SDS-PAGE, and stained with coomassie brilliant blue dye. Bands (1–6, lane 1, Figure 1A) were excised from the gel and proteins were identified by nano-LC FT-ICR mass spectrometry. Band 2 was uniquely identified as nucleolin (Ncl) (Figure 1B), and the Ncl/Tpt1 interaction was further investigated.

### Ncl/Tpt1 colocalization depends upon phosphorylation of Ncl and peaks during mitosis

**Figure 2:**
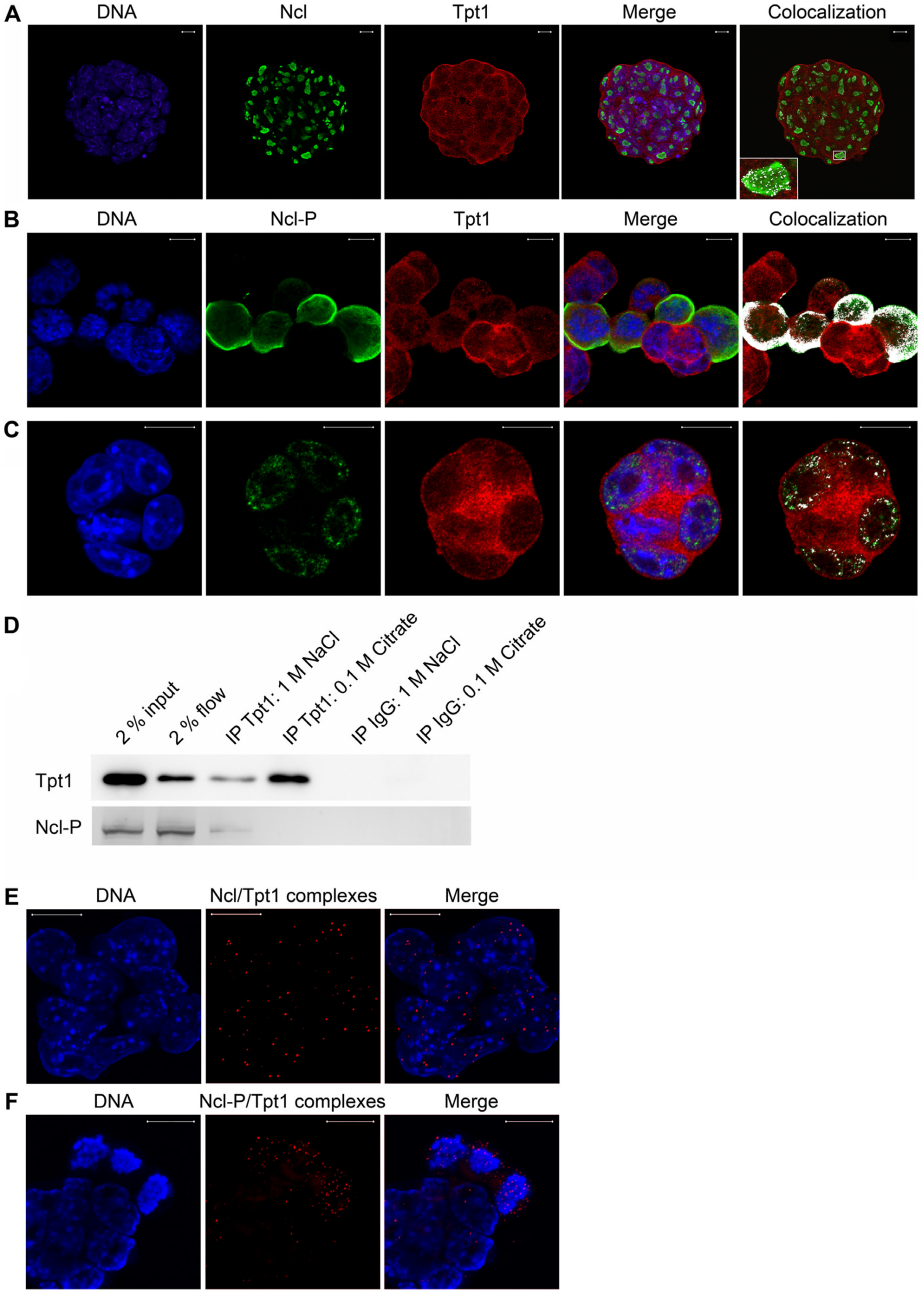
Ncl/Tpt1 interaction which peaks during mitosis and requires phosphorylations on Ncl. (A–C) Immunofluorescence confocal microscopy analysis of ES cells. DNA was counterstained with DAPI (blue). (A) ES cell colony reveals Ncl (green) nucleolar staining, whereas Tpt1 (red) localize ubiquitously in the cells. Visualization of colocalization (white) reveals that there are negligible amounts of Ncl/Tpt1 colocalization in the nucleolar compartments (see enlargement in lower left corner). (B) ES cells, arrested in metaphase by addition of demecolcine, shows that the nucleolar compartment is disassembled in mitotic cells at the same time as Ncl is phosphorylated (green) and the expression pattern more resembles Tpt1 (red) in the mitotic cells. Ncl-P/Tpt1 shows high degree of colocalization (white) during mitosis. (C) Confocal laser transmission of the green channel was increased to be able to visualize the lower amounts of Ncl-P (green) also during the interphase of the cell cycle and as shown there are some minor amounts of Ncl-P/Tpt1 colocalization (white) in this stage. (D) Co-immunoprecipitation experiments followed by Western blot analysis show that Ncl-P can be immunoprecipitated using anti-Tpt1 but not with IgG (eluted with1 M NaCl or citrate). (E–F) Immunofluorescence confocal microscopy in combination with *in situ* PLA, which is highly specific for detecting physically interacting protein-protein complexes (red). DNA was counterstained with Hoechst 33342 (blue). (E) A few Ncl/Tpt1 complexes are shown in the nucleoplasm of ES cells, in accordance with colocalization results in (D). (F) A rather high amount of Ncl-P/Tpt1 complexes are shown in the mitotic cells arrested with demecolcine, in accordance with colocalization results in (C). Scale bar represents 10 µm.

In order to examine if endogenous Tpt1 and Ncl colocalize at different stages of the cell cycle, we employed immunofluorescence analyses using confocal microscopy. Colocalization is visualized in a negligible amount in the nucleoli (Figure 2A). Since both proteins have been implicated to be important during cell division, cells were arrested in metaphase by addition of demecolcine, and stained with antibodies against phosphorylated nucleolin (anti-Ncl-P) and anti-Tpt1 (Figure 2B, see Figure S1, for comparison of anti-Ncl and anti-Ncl-P in interphase and mitotic cells, and specificity of anti-Ncl on western blot). Substantially more colocalization is visualized with Ncl-P/Tpt1 (Figure 2B–C) compared to Ncl/Tpt1 (Figure 2A). Comparison of the amount of colocalization of the Ncl-P/Tpt1 interaction in mitotic (Figure 2B) and interphase cells (Figure 2C) reveals that the colocalization is most prominent during mitosis.

To quantify the observed Ncl/Tpt1 colocalization differences between mitotic and interphase cells we calculated three different parameters: Pearson's correlation coefficient, percentage overlap of Tpt1 and percentage overlap of Ncl or Ncl-P. Tpt1 and Ncl were as expected confirmed not to colocalize (Table 1, first row) whereas Tpt1 and Ncl-P were confirmed to colocalize both in mitotic (Table 1, second row) and interphase (Table 1, third row) ES cells as indicated by good values of all three parameters but with significantly higher values for mitotic cells.

**Table 1:** Phosphorylated Ncl interacts individually with Tpt1 and Oct4.

Variables	Pearson's correlation coefficient	% overlap of Ncl or Ncl-P	% overlap of Tpt1 or Oct4
Interphase Ncl/Tpt1	0.249±0.004	10.85±0.57	5.56±0.13
Mitotic Ncl-P/Tpt1	0.761±0.013	43.44±2.65	69.66±3.05
Interphase Ncl-P/Tpt1	0.524±0.008	38.73±1.51	18.64±5.06
Interphase Ncl-P/Oct4	0.815±0.005	65.62±2.59	33.00±1.87

Ncl only interacts with Tpt1 when phosphorylated and the Ncl-P/Tpt1 interaction is most prominent during mitosis. Ncl-P also shows interaction with Oct4 during interphase. Data are represented as mean ± SEM.

In brief, Tpt1 and Ncl only colocalize when Ncl is phosphorylated and Ncl-P/Tpt1 colocaliztion peaks during mitosis.

### Ncl-P/Tpt1 colocalization decreases upon differentiation

To investigate whether Tpt1 and Ncl-P colocalize in differentiated cells, ES cells were induced to differentiate by retinoic acid treatment for up to 72 h. To obtain sufficient number of mitotic cells, demecolcine was added during the last 14 h of incubation. Undifferentiated ES cells were compared to cells treated with retinoic acid for 24, 48 and 72 h, respectively, and Pearson's correlation coefficient was found to significantly decrease after 72 h of retinoic acid induced differentiation (Table S1). These analyses imply that Tpt1 interacts with Ncl-P in ES cells, and that the Ncl-P/Tpt1 interaction decreases upon differentiation.

### Ncl-P co-immunoprecipitates with anti-Tpt1 in murine ES cells

To assess the Ncl-P/Tpt1 interaction, mitotic extracts from murine ES cells were subjected to co-immunoprecipitation with anti-Tpt1 followed by Western blot. Ncl-P was immunoprecipated with anti-Tpt1 (Figure 2D, IP Tpt1: 1 M NaCl). No co-immunoprecipitation of Ncl-P was detected in the IgG control. These findings show that Ncl-P can be immunoprecipitated together with anti-Tpt1 in mitotic murine ES cell extract, further confirming that Ncl-P interacts with Tpt1 in ES cells.

### Ncl-P physically interacts with Tpt1 in ES cells

*In situ* proximity ligation assay (PLA) [25] was used to explore the interaction between Ncl-P and Tpt1 in further detail. The distance between the primary antibodies needs to be less than 40 nm for the PLA to generate a signal, making the methodology highly specific for physically interacting protein-protein complexes, and this method works in fact very well in our cell system [24]. Anti-Ncl-P is an IgM antibody, and the PLA method works best with IgG antibodies, therefore both IgM anti-Ncl-P and two IgG anti-Ncl (recognizing both unphosphorylated and phosphorylated forms of Ncl) were used in different experiments to explore the Ncl-P/Tpt1 interaction. Quite few Ncl/Tpt1 complexes are seen in the nucleoplasm of interphase cells when using anti-Ncl (sc-8031) (Figure 2E). The second anti-Ncl (sc-13057) didn't render any Ncl/Tpt1 complexes at all (data not shown). Considerable higher amounts of Ncl-P/Tpt1 complexes are seen in the three cells arrested in metaphase by demecolcine (Figure 2F). Even though anti-Ncl-P is an IgM antibody and therefore less detected than IgG, the PLA still verifies that the Ncl-P/Tpt1 interaction occurs mostly during mitosis, which confirms the observation made by immunofluorescence analyses, and also establishes that endogenous Ncl-P physically interacts with endogenous Tpt1 especially in mitotic cells. (See Figure S2 for positive and negative controls).

### Ncl-P colcoalize with Oct4 during interphase in ES cells

**Figure 3:**
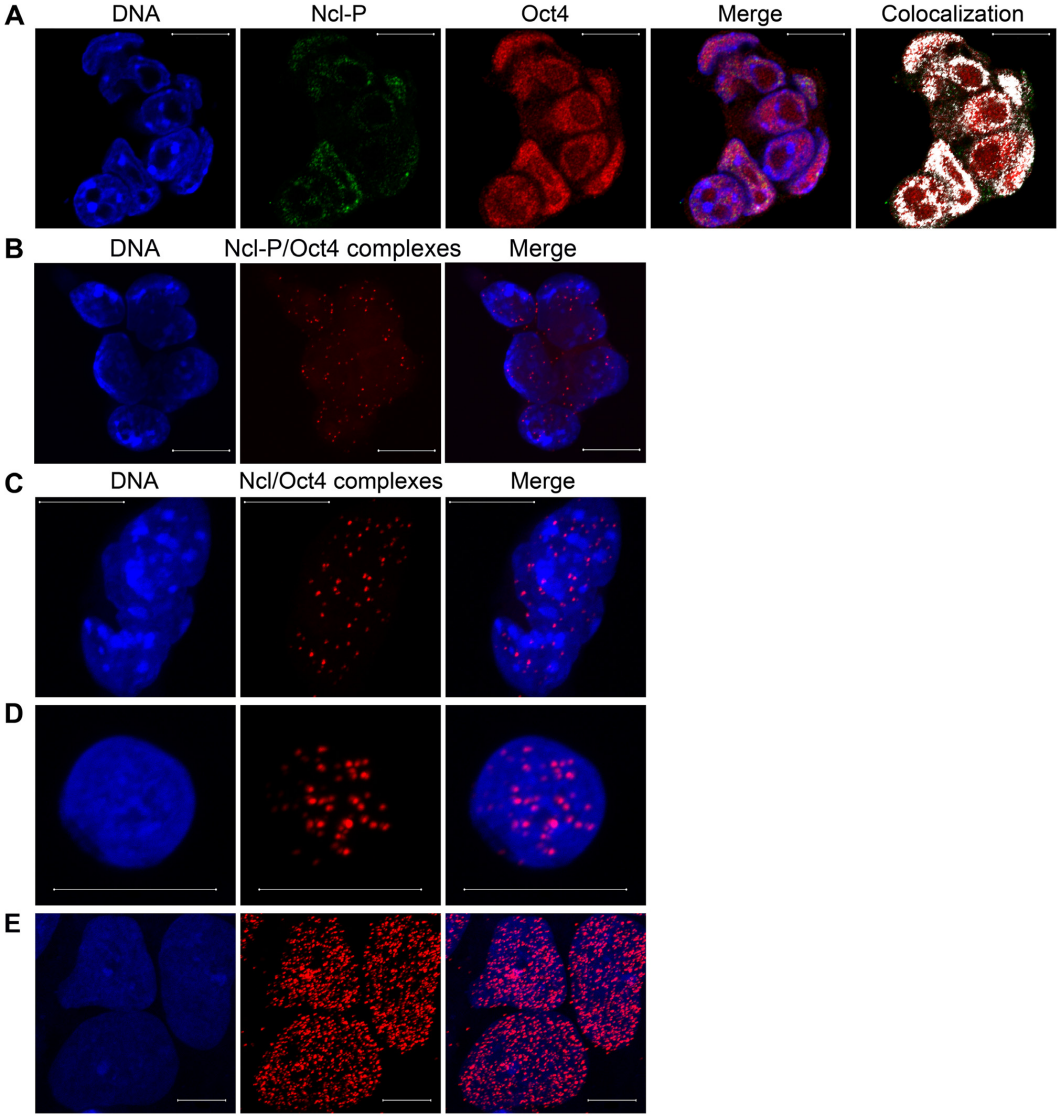
Endogenous Ncl-P/Oct4 interaction during interphase in ES cells. (A) ES cells showing similar staining of Ncl-P (green) and Oct4 (red) in the nuclei of interphase cells. Visualization of colocalization (white) reveals that Ncl-P/Oct4 colocalization is detected mostly in the nucleoplasm. DNA was counterstained with DAPI (blue). (B–E) Immunofluorescence confocal microscopy in combination with *in situ* PLA, detects Ncl-P/Oct4 complexes (red) in the nucleoplasm of both murine ES cells and hESCs. DNA was counterstained with Hoechst 33342 (blue). (B) Although using IgM anti-Ncl-P that is less detected than IgG with the *in situ* PLA, still a substantial number of Ncl-P/Oct4 complexes is observed in the nucleoplasm of murine ES cells,which confirm their interaction. (C) Using anti-Ncl (recognizing both unphosphorylated and phosphorylated forms of Ncl) the same complex pattern of Ncl/Oct4 complexes is seen spread out in the nucleoplasm of murine ES cells. (D) One small hESC with a number of Ncl/Oct4 complexes in the nucleoplasm confirms that the interaction also exists in hESCs. (E) Three larger hESCs, probably cells that have started to differentiate, show really high amounts of Ncl/Oct4 complexes in the nucleoplasm, while no complexes are observed in the nucleoli (seen as empty round spaces in the cells), confirming that Ncl needs to be phosphorylated to interact also with Oct4. Scale bar represents 10 µm.

The staining pattern of Ncl-P in interphase cells (Figure S1B) shows very similar staining pattern as the stem cell marker Oct4, and since both proteins are involved in transcription we investigated if they interact in ES cells. In order to examine if endogenous Ncl-P and Oct4 colocalize, we employed immunofluorescence studies using confocal microscopy. High colocalization is detected in the nucleoplasm (Figure 3A). To quantify that the colocalization is significant we analyzed the cells with the BioPix iQ 2.0 software. Table 1 (fourth row) indicates really good colocalization with both a high Pearson's correlation coefficient and also good percentage overlap from both proteins. These analyses indicate that Oct4 and Ncl exist in a complex in murine ES cells when Ncl is phosphorylated, and show really prominent pattern of interaction in interphase ES cells.

### Oct4 physically interacts with Ncl-P in both human and murine ES cells

To further explore the endogenous interaction between Oct4 and Ncl-P *in* situ PLA was used. Both anti-Ncl and anti-Ncl-P were tested in different experiments. With anti-Ncl-P reasonable number of Ncl-P/Oct4 complexes are seen in the nucleoplasm of the interphase murine ES cells (Figure 3B), and the same complex pattern was observed with anti-Ncl (sc-13057) (Figure 3C) while hardly any signal was rendered using anti-Ncl (sc-8031) (data not shown). All together this confirms the immunofluoresence results and furthermore proves that Ncl-P/Oct4 physically interact in ES cells.

Next we asked the question if the interaction is specific to murine ES cells or if it also exists in human ES cells (hESC). Since Oct4 showed no difference in complex formation between anti-Ncl-P and anti-Ncl in murine ES cells, together with that *in situ* PLA works best with IgG antibodies, we used anti-Ncl (sc-13057) to examine the interaction between Ncl-P/Oct4 in hESCs. Figure 3D shows one small hESC with quite many Ncl/Oct4 complexes, indicating that the interaction also exists in hESCs. The most interesting observation is that in hESCs that have started to spontaneously differentiate (cells are growing bigger) have really high amount of Ncl/Oct4 complexes (Figure 3E), implying that the interaction may be important during initial differentiaiton. (See Figure S2, for positive and negative controls for *in situ* PLA). This observation could not be seen in murine ES cells, even though ES cell cultures contain some cells that have started to spontaneously differentiate. All three cells visualized in Figure 3E lack red dots in the compartments that corresponds to the nucleoli, strengthening our finding that Oct4 only interacts with Ncl when phosphorylated, since Ncl-P are restricted to the nucleoplasm of interphase cells, while unphosphorylated Ncl are restricted to the nucleoli (Figure S1B).

To conclude, Oct4 and Ncl-P interact in interphase cells in both human and murine ES cells and the interaction increases during spontaneous differentiation of hESCs.

## Discussion

The data presented here reveals new protein-protein complexes involving Ncl in murine and human ES cells, where we have identified Oct4 and Tpt1 as two endogenous Ncl-P interaction partners. All experiments with murine ES cells were performed on two different ES cell lines (R1 and RW4) in parallel. We found no discernible differences between the ES cell lines, and one experiment also included human ES cells, indicating our findings to be generally applicable for ES cells, and not species or cell line specific.

In our previous search for Tpt1 interaction partners in ES cells [24] we found that Tpt1 forms a complex with Npm1. In the same screen additional potential interaction partners were found. One of the factors found were actin, which has recently been reported to interact with Tpt1 [26] and we have now also revealed that Ncl-P forms a complex with Tpt1 in murine ES cells. Ncl has previously been reported to be expressed at high levels in both murine [15] and human ES cells [16] where high levels of Tpt1 also were reported. Recent proteome analysis of mouse ES cell lines has furthermore revealed that the presence of Tpt1 is a characteristic of undifferentiated ES cells [27]. Tpt1 is well conserved and expressed in all eukaryotic organisms but no main function has yet been found.

Accumulating evidence demonstrate that Tpt1 and Ncl are important for cell proliferation. *Tpt1* is up-regulated during the entry into the cell cycle. Over-expression of *Tpt1* results in slow growing cells and a delayed cell cycle progression [24], [28] whereas over-expression of a Tpt1 double mutant, in which the two Plk1 phosphorylation sites have been substituted for alanines, induced a dramatic increase in multinucleated cells, rounded cells with condensed ball-like nuclei, and cells undergoing cell death [29]. Down-regulation experiments using RNA-interference have shown that Ncl is required for correct mitosis, given that absence of Ncl gave a prolonged cell cycle with misaligned chromosomes and defects in spindle organization [18]. Similar experiments revealed that absence of Ncl forced cells into growth arrest, accumulated in G2 phase, but also resulted in increased apoptosis and effects on the nucleus as well as defects in centrosome duplication [19].

According to the above we reasoned that the found interaction between Ncl-P/Tpt1, which peaks during mitosis, plays a part in cell proliferation or cell cycle regulation. This is further supported by that we found the colocalization to decrease upon retinoic acid induced differentiation, since differentiated cells proliferate much slower than ES cells.

We previously found an interaction between Tpt1 and Npm1 [24] that also showed a cell cycle dependent pattern of interaction with a significant peak during mitosis. Ncl and Npm1 have previously been shown to be interaction partners [30]. One could speculate if all three proteins may exist in a complex during mitosis, but we do not think this is the case. Npm1/Ncl was previously shown not to interact during prometaphase and metaphase [30], phases where we detect individual interactions of Ncl-P/Tpt1 and Tpt1/Npm1, suggesting that they are not part of a single complex.

Remarkably, we found that Ncl-P also forms a complex with Oct4 and we observe this complex during interphase in both murine and human ES cells. Oct4 (also denoted Oct3, Pou5f1, and NF-A3) belongs to the POU transcription factor family. POU transcription factors were originally identified as DNA-binding proteins that are able to activate the transcription of genes containing an octameric sequence called the octameric motif (AGTCAAAT), within the promoter or enhancer region. Oct4 has been proven to be essential for the identity of the pluripotential founder cell population in the mammalian embryo [5]. Its endogenous expression is normally tightly restricted to embryonic stem and germ cells, and illegitimate *Oct4* activation is also typical for embryonic carcinoma cells [5], [31]. The precise levels of Oct4 expression was reported to determine the fate of ES cells to either differentiate, dedifferentiate or self-renew [6]. Oct4 has also been reported to be a potent nuclear reprogramming factor, where the Oct4 distribution and level in mouse clones showed consequences for pluripotency and the development of the somatic cell clones [7]. With the newest method to reprogram somatic cell nuclei through retroviral introduction by different sets of transcription factors to create iPS cells, Oct4 has again been proven to be one of the necessary components for successful nuclear reprogramming [1], [2], [3], [4].

Ncl has been described to interact with or be a component of several transcription factor complexes, one of them being the B cell-specific transcription factor and switch region binding protein, LR1 [22]. LR1 has previously been shown to regulate c-*myc* transcription in B-cell lymphomas [32] and Ncl has independently been found to be a c-*myc* target gene, were the c-Myc oncoprotein induces the levels of Ncl [33]. The LR1 binding site (CCTCCTGGTCAAGGCTGAA) shows some interesting similarities to the octameric motif, with five of the eight bases being exactly the same, and the base proceeding and descending these five bases can be altered without LR1 losing its binding activity [34]. Even more interesting is that LR1-binding activity was also reported to be dependent on phosphorylations of LR1 [35] and our found interaction between Oct4 and Ncl is only occurring when Ncl is phosphorylated, indicating a similar mechanism for Ncl-P/Oct4 as components of a transcription factor complex in ES cells. All the above implies that Ncl-P is a component of a transcription factor complex together with Oct4 in ES cells. Although further investigations are required to verify this, by for example chromatin immunoprecipitation on sequences where Oct4 are known to bind, although none of our anti-Ncl works on immunoprecipitation so unfortunately we have not been able to perform the necessary experiments and can only speculate about it. Most interesting is the observed increase of Ncl-P/Oct4 complexes in spontaneously differentiated hESCs. It is tempting to speculate that Ncl-P works as a repressor to the activity of Oct4, by forming complexes with it when the ES cells start to differentiate. This might either allow transcription of genes repressed by Oct4 in ES cells or inhibit ES cell specific transcription.

To conclude, we have identified two new interaction partners to Ncl in ES cells, both interactions occurring when Ncl is phosphorylated. Ncl-P/Tpt1 interact most prominently during mitosis and the interaction decreases upon retinoic acid induced differentiation; this indicates of a roll in cell proliferation or cell cycle regulation of rapidly dividing cells. Ncl-P/Oct4 interact in the nucleoplasm of interphase cells in both murine and human ES cells and show increased interaction in the beginning of spontaneously differentiated human ES cells. Similarities are found in the literature of Oct4 and Ncl binding sites as transcription factors, highly proposing Ncl-P to be together with Oct4 in a transcription factor complex in ES cells and that the complex formation is increasing during the initiation of spontaneous differentiation. Regarding our data here we propose Ncl-P as a connecting link between proliferation and transcription. Expression of Ncl-P may therefore improve reprogramming of somatic cells.

## Materials and Methods

### Tpt1 interaction partner screen

Plasmid construction, expression and purification of recombinant Tpt1, preparation of sepharose-linked Tpt1 and screen for new interaction partners with ES cell extracts was performed according to previously published material [24].

### Cell culture

Cell lines were grown at 37°C in a humidified atmosphere containing 5% CO_2_. Murine ES cell lines RW4 [36] (derived from 129X1/SvJ) and R1 [37] (derived from 129X1 x 129S1) were maintained on mitomycin C inactivated mouse embryonic fibroblasts in Dulbecco's modified eagle's medium supplemented with 15% fetal calf serum, 1.0 mM sodium pyruvate, 0.1 mM nonessential amino acids, 2.0 mM L-glutamine, 0.1 mM β-mercaptoethanol, 100 U/100 µg penicillin/streptomycin, 20 mM HEPES pH 7.3 and 1000 U/ml leukemia inhibitory factor. Human ES cell line SA461 [38] was cultured and provided from Cellartis AB (Göteborg, Sweden).

### Cell extracts

Whole cell extracts were prepared by harvesting confluent cell cultures containing approximately 3×10^7^ cells. Harvested cells were incubated in ice cold extraction buffer (10 mM Tris-HCl pH 7.4, 10 mM MgCl_2_, 10 mM KCl, 1.0 mM DTT) containing protease inhibitor cocktail tablet (Complete, Roche Diagnostics) for 10 min, 4°C. The addition of NP40 to 1% (v/v) was followed by incubation for 10 min, 4°C. Cell lysates were homogenized and NaCl was added to a final concentration of 420 mM followed by incubation for 1 h, 4°C. The extracts were cleared by centrifugation (19,000x*g*, 1 h, 4°C) and the supernatant was frozen and stored in liquid nitrogen.

### Western blot

Proteins were separated using SDS-PAGE, followed by semi-dry electrotransfer onto polyvinylidene difluoride membranes for 1 h, 100mA/gel in transfer buffer (48 mM Tris, 39 mM Glycin, 1.3 mM SDS, 10% methanol) and immunologically detected. Membranes were blocked with 5% skim milk in phosphate-buffered saline (PBS) containing 0.1% Tween 20 for 1 h and incubated with primary antibody (anti-TCTP [ab37506, Abcam], anti-TG3 against phosphorylated nucleolin [kind gift from Peter Davies]) in blocking solution overnight at 4°C. After washing with PBS-Tween, blots were incubated with secondary antibody (AP conjugated goat anti-mouse IgM+IgG+IgA (H+L) [Southern Biotechnology Associates] or HRP-conjugated anti rabbit IgG [NA934VS, Amersham Biosciences]) in blocking solution for 1 h at room temperature. Visualization of proteins was done with BCIP/NBT kit (Invitrogen) or Pierce ECL Western Blotting Substrate (Nordic Biolabs).

### Co-immunoprecipitation

Co-immunoprecipitation was performed with Dynabeads Protein G (Invitrogen) according to the manufacturer's protocol by addition and crosslinking with dithiobispropionimidate-2HCl of 10 µg polyclonal anti-TCTP [ab37506, Abcam] or normal IgG antibody [sc-2027, Santa Cruz]. Mitotic extracts were prepared by arresting ES cells in metaphase by adding Demecolcine (0.02 µg/ml, Invitrogen) 14 h prior to extract preparation. Mitotic extracts were incubated with the antibody-beads over night, 4°C. Proteins were eluted in 1 M NaCl (50 mM Tris pH 7.5, 1.0 M NaCl, 0.1% NP40, 1.0 mM DTT) with the use of the magnet. Extended elution, where antibodies were eluted, was done with 0.1 M Citrate with the use of the magnet. Elutes were mixed with 2x Laemmli buffer and heated to 95°C for 5 min and analyzed by Western blot.

### Immunofluorescence analyses and confocal microscopy

5–8×10^4^ cells were grown on glass coverslips overnight. Metaphase arrest was achieved by incubation with 0.02 µg/ml demecolcine (Invitrogen) for 14 h and differentiation was achieved by incubation with 2.0 µM retinoic acid for up to 72 h. Cells were fixed in 4% paraformaldehyde/PBS for 20 min, permeabilized with 0.25% Triton X-100/PBS for 5 min, and blocked in 5% normal goat serum or 10% fetal calf serum in 0.1% Triton X-100/PBS for 20 min. Primary (anti-HRF/TPT1 [Code No. M099-3, Clone 6E9, Nordic Biosite]; anti-C23 [sc-13057 or sc-8031, Santa Cruz]; anti-Oct-3/4 (H-134): sc-9081 [Santa Cruz]; anti-Oct4 [611203, Clone 40, BD Biosciences]; anti-TG3 against phosphorylated nucleolin [kind gift from Peter Davies]) and secondary (Alexa Fluor 488 or 555 labeled goat anti-mouse IgG or IgM; Alexa Fluor 488 or 555 labeled goat anti-rabbit IgG [Molecular Probes]) antibodies diluted in 0.1% Triton X-100/PBS/0.5% normal goat serum or 1% fetal calf serum were sequentially added for 2 and 1 h, respectively, each followed by washes in 0.1% Triton X-100/PBS. Nuclei were counterstained with 4,6-diamidino-2-phenylidole (DAPI). Coverslips were air-dried, mounted, and analyzed using an inverted Zeiss LSM 510 META confocal microscope equipped with a Zeiss image processing system. An 63×/1.4 oil NA objective and sequential scanning with narrow band-pass filters was used (BP 420–480 for DAPI, 505–530 for Alexa 488 and BP 560–615 for Alexa 555).

### Colocalization analysis

Colocalization was visualized using ImageJ (http://rsb.info.nih.gov/ij). Confocal micrographs were collected at 0.38 µM intervals to create z-axis stacks, and images rendered from the z-axis stacks were analyzed with the BioPix iQ 2.0 software. Mitotic and interphase cells were selected manually. A minimum of five different z-axis stacks, containing at least 10 cells each, were taken for each analysis. Data are represented as mean±SEM (standard error of mean). SPSS 13.0 was used for statistical evaluation of colocalization results generated by BioPix iQ 2.0. One-way analysis of variance followed by post hoc and Turkey's test, were done to evaluate significant changes between groups. Values are considered significant if p ≤ 0.01 for the differences between analyzed groups.

### Generic *in situ* proximity ligation assay

3−4×10^4^ cells were grown on chamber slides overnight. Fixation, permeabilization, blockage and primary antibody (anti-HRF/TPT1 [Code No. M099-3, Clone 6E9, Nordic Biosite]; HRF (FL-172): sc-30124 [Santa Cruz]; anti-C23 [sc-13057 or sc-8031, Santa Cruz]; anti-Oct-3/4 (H-134): sc-9081 [Santa Cruz]; anti-Oct4 [611203, Clone 40, BD Biosciences]; anti-TG3 against phosphorylated nucleolin [kind gift from Peter Davies]) incubation were performed as decribed for immunofluorescence analyses. Duolink (Olink Biosciences) *in situ* proximity ligation assay (PLA) was performed according to the manufacturer's protocol. PLA probes were diluted in 0.1% Triton X-100/PBS/1% fetal calf serum and incubated in a pre-heated humidity chamber for 1 h at 37°C, followed by hybridization, ligation, amplification and detection. Nuclei were counterstained with Hoescht 33342. Slides were analyzed by confocal microscopy.

## Supporting Information

Figure S1 Endogenous Ncl vs. Ncl-P in ES cells. (A) ES cells arrested at metaphase by addition of demecolcine solution. Ncl (green) is mostly visible in the nucleolar compartment; meanwhile the Ncl-P (red) is highly expressed only in the cells that are arrested in mitosis. (B) Confocal laser transmission of the red channel was increased to be able to visualize the lower amounts of Ncl-P (red) in the interphase cells. Compared to Ncl (green), Ncl-P shows no staining in the nucleolar compartment, but is visualized in the nucleoplasm, best visualized in the merge picture. DNA was counterstained with DAPI (blue). Scale bar represents 10 µm. (C) Western blot showing the specificity of anti-Ncl (sc-13057), only giving one band in the right size (approximately 110 kDa).
(3.87 MB TIF)

Figure S2 *In situ* proximity ligation assay controls. (A-B) Positive control for *in situ* PLA. Immunofluorescence confocal microscopy in combination with *in situ* PLA, detects Npm1/Ncl complexes (red) in both murine ES cells (A) and hESCs (B). Ncl/Nmp1 was used as a positive control since their interaction has been demonstrated previously [Liu, H. T., and Yung, B. Y. (1999) *In vivo* interaction of nucleophosmin/B23 and protein C23 during cell cycle progression in HeLa cells. Cancer Lett 144, 45-54]. (C-E) Negative controls for murine ES cells (C & E) and hESCs (D) to visualize *in situ* PLA background staining when no primary antibodies were used (C-D) and Nanog-Ncl (E), which do not interact in ES cells. A few red dots did appear but in consideration of the amount obtained in the experiments, it can be neglected. DNA was counterstained with Hoechst 33342. Scale bar represents 10 µm.
(10.18 MB TIF)

Table S1 (0.04 MB DOC)
