# Supplementary figures and images for: Phosphorylated Nucleolin Interacts with Translationally Controlled Tumor Protein during Mitosis and with Oct4 during Interphase in ES Cells
Source: PLoS One. 2010 Oct 27;5(10):e13678. doi: 10.1371/journal.pone.0013678 (PMC2965110; doi:10.1371/journal.pone.0013678)

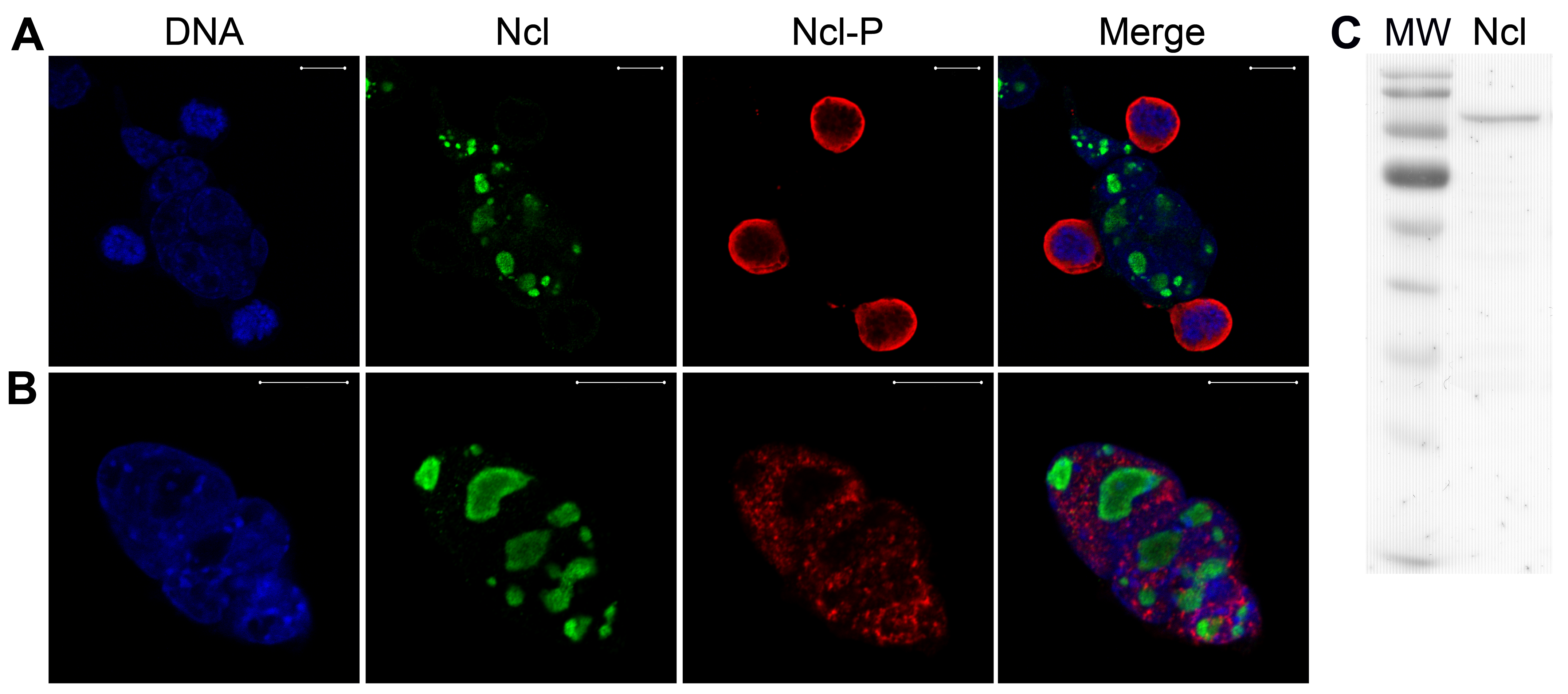

Supplement: Figure S1 — Endogenous Ncl vs. Ncl-P in ES cells. (A) ES cells arrested at metaphase by addition of demecolcine solution. Ncl (green) is mostly visible in the nucleolar compartment; meanwhile the Ncl-P (red) is highly expressed only in the cells that are arrested in mitosis. (B) Confocal laser transmission of the red channel was increased to be able to visualize the lower amounts of Ncl-P (red) in the interphase cells. Compared to Ncl (green), Ncl-P shows no staining in the nucleolar compartment, but is visualized in the nucleoplasm, best visualized in the merge picture. DNA was counterstained with DAPI (blue). Scale bar represents 10 µm. (C) Western blot showing the specificity of anti-Ncl (sc-13057), only giving one band in the right size (approximately 110 kDa). (3.87 MB TIF) [file pone.0013678.s001.tif]

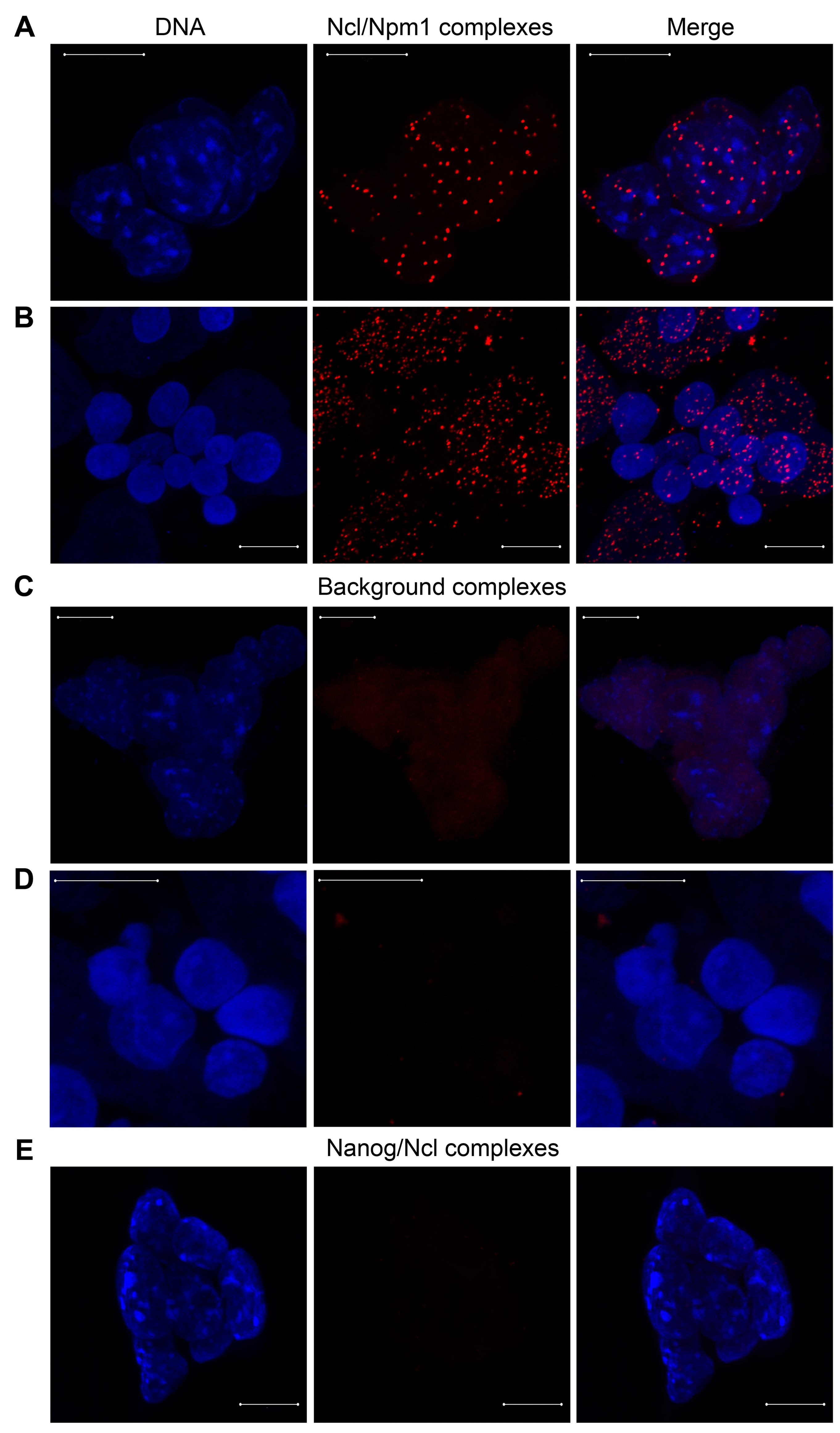

Supplement: Figure S2 — In situ proximity ligation assay controls. (A-B) Positive control for in situ PLA. Immunofluorescence confocal microscopy in combination with in situ PLA, detects Npm1/Ncl complexes (red) in both murine ES cells (A) and hESCs (B). Ncl/Nmp1 was used as a positive control since their interaction has been demonstrated previously [Liu, H. T., and Yung, B. Y. (1999) In vivo interaction of nucleophosmin/B23 and protein C23 during cell cycle progression in HeLa cells. Cancer Lett 144, 45-54]. (C-E) Negative controls for murine ES cells (C & E) and hESCs (D) to visualize in situ PLA background staining when no primary antibodies were used (C-D) and Nanog-Ncl (E), which do not interact in ES cells. A few red dots did appear but in consideration of the amount obtained in the experiments, it can be neglected. DNA was counterstained with Hoechst 33342. Scale bar represents 10 µm. (10.18 MB TIF) [file pone.0013678.s002.tif]
