# Supplementary material for: Phosphorylated Nucleolin Interacts with Translationally Controlled Tumor Protein during Mitosis and with Oct4 during Interphase in ES Cells
Source: PLoS One. 2010 Oct 27;5(10):e13678. doi: 10.1371/journal.pone.0013678 (PMC2965110; doi:10.1371/journal.pone.0013678)
Supplement: Table S1 — (0.04 MB DOC) [file pone.0013678.s003.doc]

**Table S1. RA induced differentiation decreases Ncl-P/Tpt1 colocalization**

Data are presented as mean±SEM.

Significant statistical difference with p≤ 0.01 is indicated by *, n.s.= not significant
